# A wolf in sheep’s clothing: The description of a fly resembling jumping spider of the genus *Scoturius* Simon, 1901 (Araneae: Salticidae: Huriini)

**DOI:** 10.1371/journal.pone.0190582

**Published:** 2018-01-24

**Authors:** Robert Perger, Gonzalo D. Rubio

**Affiliations:** 1 Colección Boliviana de Fauna, La Paz, Bolivia; 2 National Research Council of Argentina (CONICET), Experimental Station of Agriculture (EEA-INTA), Misiones, Argentina; Charles University, CZECH REPUBLIC

## Abstract

Fly resemblance in arthropods is much less common than e.g., resemblance to ants or wasps, and has been mainly observed in beetles. Putative fly mimicry in arachnids has been reported only from the jumping spider genus *Saitis*. However, recent literature has attributed the fly-resembling characteristics in *Saitis* to sexual signalling during courtship. The lack of observation of fly mimicry in spiders is not surprising as flies belong to the most important prey item group of spiders. In this study, a new fly-resembling species of the jumping spider tribe Huriini, *Scoturius dipterioides* **sp. nov**., from the pre-Andean Chiquitano forest at the Bolivian orocline is described and illustrated. The new species was tentatively placed into *Scoturius* because the epigynum has a single large elliptical opening. *Scoturius dipterioides* **sp. nov**. is distinguished from all other species of this tribe by a combination of following characteristics: epigynum with a large anterior elliptical atrium and initial portion of the copulation ducts dilated as a chamber (shared with *Urupuyu*); relatively joined copulation openings and short copulation ducts; kidney-shaped spermathecae, advanced at the atrium level. Several somatic features, two of them found exclusively in *S*. *dipterioides* **sp. nov**., increase the resemblance to flies. The Huriini are currently the only spider tribe that is suggested to feature fly mimics.

## Introduction

Fly resemblance in arthropods is much less frequent than e.g., resemblance to ants or wasps, and has been mainly found in beetles [1]. The selective advantage of imitating flies is not clear because flies are not known for being protected by any weaponry that may repel predators [1]. One possible explanation for the selection of a fly-like appearance is that such forms may gain protection because flies are sufficiently hard to catch that visually-oriented predators, like birds, sometimes decline the opportunity to pursue them [1]. However, empirical data is still necessary to support this hypothesis [2].

Jumping spiders (Salticidae) are present in all non-polar terrestrial ecosystems and represent the most diverse family of modern spiders with more than 5800 species described [3]. The most astonishing and distinguishing characteristic of salticid spiders lies in the evolution of their highly acute vision, particularly with respect to their large, tubular principal eyes [4]. Based on this feature, jumping spiders are probably an important selective pressure shaping the evolution of diurnal arthropods that perch on vegetation [5]. Jumping spiders may be responsible for selecting various mimicry types, for example jumping spider mimicry in tephritid flies, fulgoroid homopterans and lepidopterans [6]. Given that jumping spiders prey on other spiders as well, including representatives of their own family, they may also have fostered the evolution of several mimicry types within spiders themselves [5]. Spiders are recognized for resembling to a wide range of insects, such as termites (resembled by Zodariidae) [7], mutilid wasps (resembled by Salticidae) (e.g. [8]), caterpillars (resembled by Theridiidae) [9], or beetles (resembled by Eresidae and Salticidae) (e.g. [10]). However, resemblance to ants is by far the most recurrent among spiders [11]. In jumping spiders, the resemblance to ants (or wasps) has evolved at least 12 times [12].

Fly resemblance in arachnids has been reported only from one single case. Morrison [13] suggested that the males of *Saitis* (Salticidae) wave their elongated, thick and brightly ornamented third pair of legs to resemble to flies. However, more recent literature [14,15] has attributed the shape and movements of the legs to sexual signalling during courtship. The lack of observation of fly resemblance in spiders is not surprising as flies belong to the most important prey item group of spiders.

Huriini Simon, 1901, is a tribe of the Salticidae including six genera with 16 species [12] that are seen exclusively in South America and defined by the combined presence of the following traits: small chelicerae with three to five teeth on the promargin and one tooth on the retromargin; the fourth leg much longer than the third; the third ocular row wider or at least equal to the first row; a narrow clypeus and two or three tibial retrolateral apophyses [16,17].

The monotypic genus *Scoturius* Simon, 1901 is distinguished from the other five genera of the Huriini by three tibial apophyses, the bulb without middle apophysis and epigynum with large elliptical opening [16]. The sole species of *Scoturius*, *S*. *tigris* Simon 1901, has been reported from Brazil (states of Minas Gerais and Mato Grosso), Argentina (Misiones province) and Paraguay (Asunción) [16].

In this contribution, an unknown species of the genus *Scoturius* from the pre-Andean Chiquitano forest at the Bolivian orocline is described, and for the first time, potential fly mimicry is reported in the Huriini, representing the only known case of a fly-resembling form in spiders.

## Material and methods

Permits for field work were granted by the Ministerio de Medio Ambiente y Agua, Bolivia. Permission to utilize the field site was obtained from the owners. This study did not involve any endangered or protected species.

**Fig 1:**
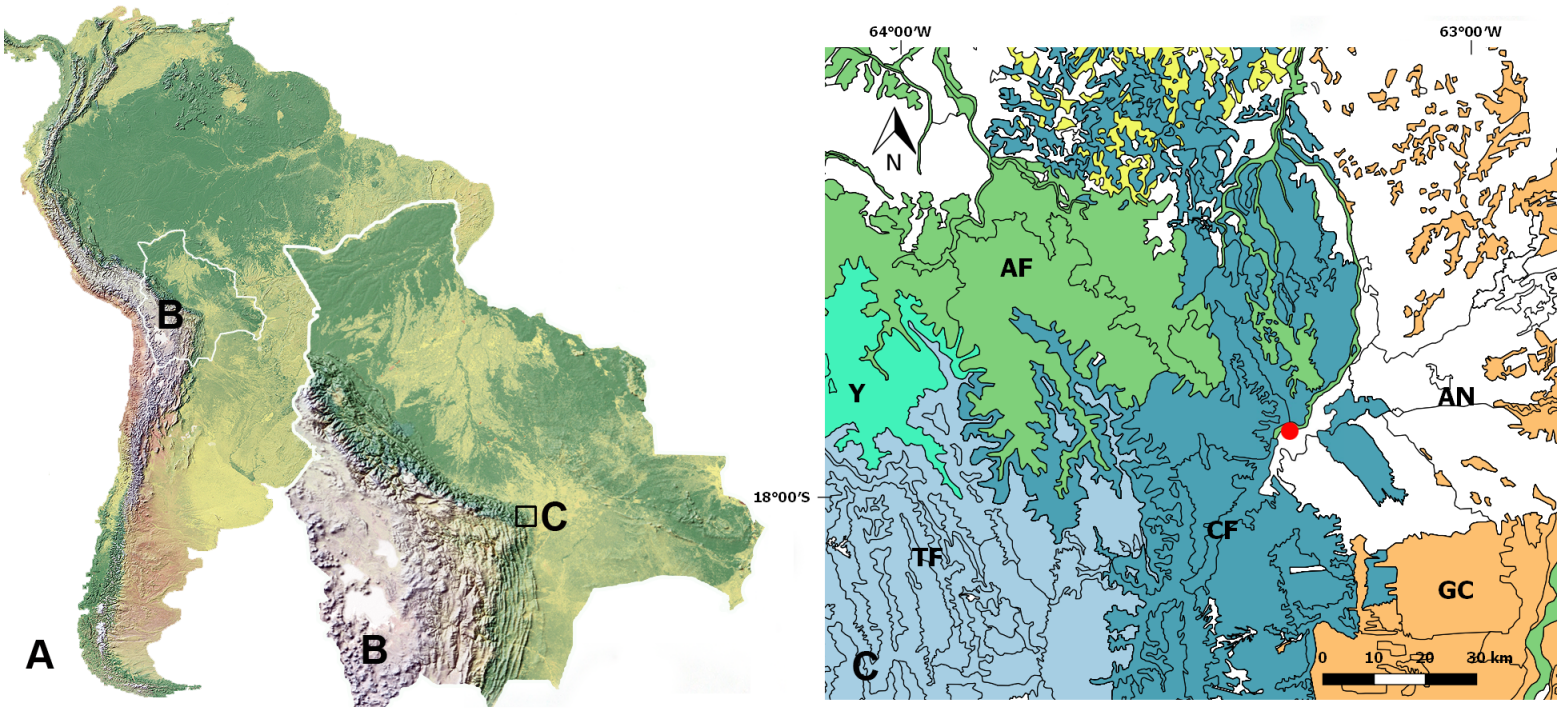
Study area and collection location of *Scoturius dipterioides* sp. nov. A. South America. B. Bolivia; the area of the Bolivian orocline indicated by black rectangle; map derived from Natural Earth World physical map with shaded relief at 1.24 km resolution (Natural Earth (public domain): http://www.naturalearthdata.com). C. Ecosystems at the Bolivian orocline, Santa Cruz department, Bolivia; map derived with the shapefile of the biogeographical regionalization by Navarro & Ferreira [19], using the Geographic Information System QGIS 2.14 (available at http://www.qgis.org/en/site/), study area indicated by red point. AN. anthropogenic habitat. AF. Amazon rainforest. CF. Chiquitano forest. GC. Gran Chaco forest. TF. Bolivian Tucuman forest. Y. Bolivian Yungas forest.

A fragment of primary forest (17°52′59S; 63°19′04W) of ~10 ha at the base of the Bolivian orocline at an elevation of 480 meters was surveyed (Fig 1). The forest fragment is situated in the Andrés Ibáñez province, between the city of Santa Cruz de la Sierra (distance to the western limit ~7 km), La Guardia village (distance to the eastern limit ~500 m) and Piraí River (distance ~600 m). It is one of the few scattered forest fragments that remain in the mosaic of urbanizations and agricultural plains this side of the Piraí River. The average annual precipitation in the study area is 1085 mm with a mean temperature of 24.1°C [18]. According to the biogeographical regionalization of Navarro & Ferreira [19], the ecosystem in the study location is considered mesophytic-phreatophytic Chiquitano forest of the alluvial plains of Santa Cruz department (Fig 1C). Tree indicator species for this ecosystem are *Albizia niopoides* (Spruce ex Bentham) Kuntze and *Gallesia integrifolia* (Sprengel) Harms [19]. The study area is located ~600 m away from riparian Amazon whitewater forest, which runs along Piraí River. Additionally, the ecosystem may be influenced by the relatively close Andean forests in the west and subtropical deciduous Gran Chaco forests in the south (see Fig 1C).

Sampling was performed from November of 2015 to January 2017 at least once a month, totalling 100 hrs. During one sampling event, two individuals of an unknown jumping spider species were collected from mid-storey branches via beating-tray sampling and retained our attention according to their particular morphology, which is subsequently reported. In order to investigate the presence of potential fly models of the new spider species, the beating tray was placed close to the tree from which the two spider individuals were collected, and flies with a grey-blackish body pattern that landed on the white sheet were photographed. Only flies having about the same body length as the largest of both spiders (3.9 mm) were considered. The body length of the flies was approximated on the photographs by using the pattern of the white sheet as a reference (a subjective estimation).

Morphological terms and description formats follow the main recent studies on similar jumping spiders [17]. Female genitalia were dissected as in Levi [20], examined after digestion in hot ~15% KOH solution (details in [21]) and clarified in clove oil to examine the internal structures. Temporary preparations were observed and photographed using a Leica DM500 compound microscope and a Leica M60 stereomicroscope. Sexual structures were sketched on incident light photograph models using a camera lucida. All measurements, which were obtained with an ocular micrometre following Ruiz and Maddison [17], are given in millimetres. The following abbreviations are used in chaetotaxy description: d, dorsal; p, prolateral; r, retrolateral; v, ventral. Photographs of live spiders were taken with a digital compact Canon SX700HS camera. Photographs of preserved specimens were taken with a Visionary Digital Passport II Imaging system at the Zoological Museum, University of Hamburg, Germany. The coordinates and a shapefile of the biogeographical regionalization by Navarro & Ferreira [19] were visualized using the geographic information system, QGIS (version 2.14.3, http://www.qgis.org/en/site/).

The examined specimens were deposited at the arachnological collection of the Instituto de Biología Subtropical, Misiones (IBSI-Ara, G. Rubio). One leg of the female holotype was used for extraction of tissue samples for future studies of DNA (indicated in the text as “tiss.s.GDR”), and was also deposited at the IBSI.

The following abbreviations are used: AME, anterior median eyes; ALE, anterior lateral eyes; PLE, posterior lateral eyes; PME, posterior median eyes (the “small eyes”).

### Nomenclatural acts

The electronic edition of this article conforms to the requirements of the amended International Code of Zoological Nomenclature, and hence the new names contained herein are available under that Code from the electronic edition of this article. This published work and the nomenclatural acts it contains have been registered in ZooBank, the online registration system for the ICZN. The ZooBank LSIDs (Life Science Identifiers) can be resolved and the associated information viewed through any standard web browser by appending the LSID to the prefix “http://zoobank.org/”. The LSID for this publication is: urn:lsid:zoobank.org:pub: F987E1B8-E743-49F5-AD32-C63053CC02F7. The electronic edition of this work was published in a journal with an ISSN, and has been archived and is available from the following digital repositories: PubMed Central, LOCKSS.

## Results

Taxonomy

**Family Salticidae Blackwall, 1841**

**Subfamily Salticinae Blackwall, 1841**

**Tribe Huriini Simon, 1901**

**Genus *Scoturius* Simon, 1901**

*Scoturius* Simon 1901: 584, 585 (n. gen.); Petrunkevitch 1928: 203; Roewer 1954: 1184; Bonnet 1958: 3976; Brignoli 1983: 628; Galiano 1987: 287.

**Fig 2:**
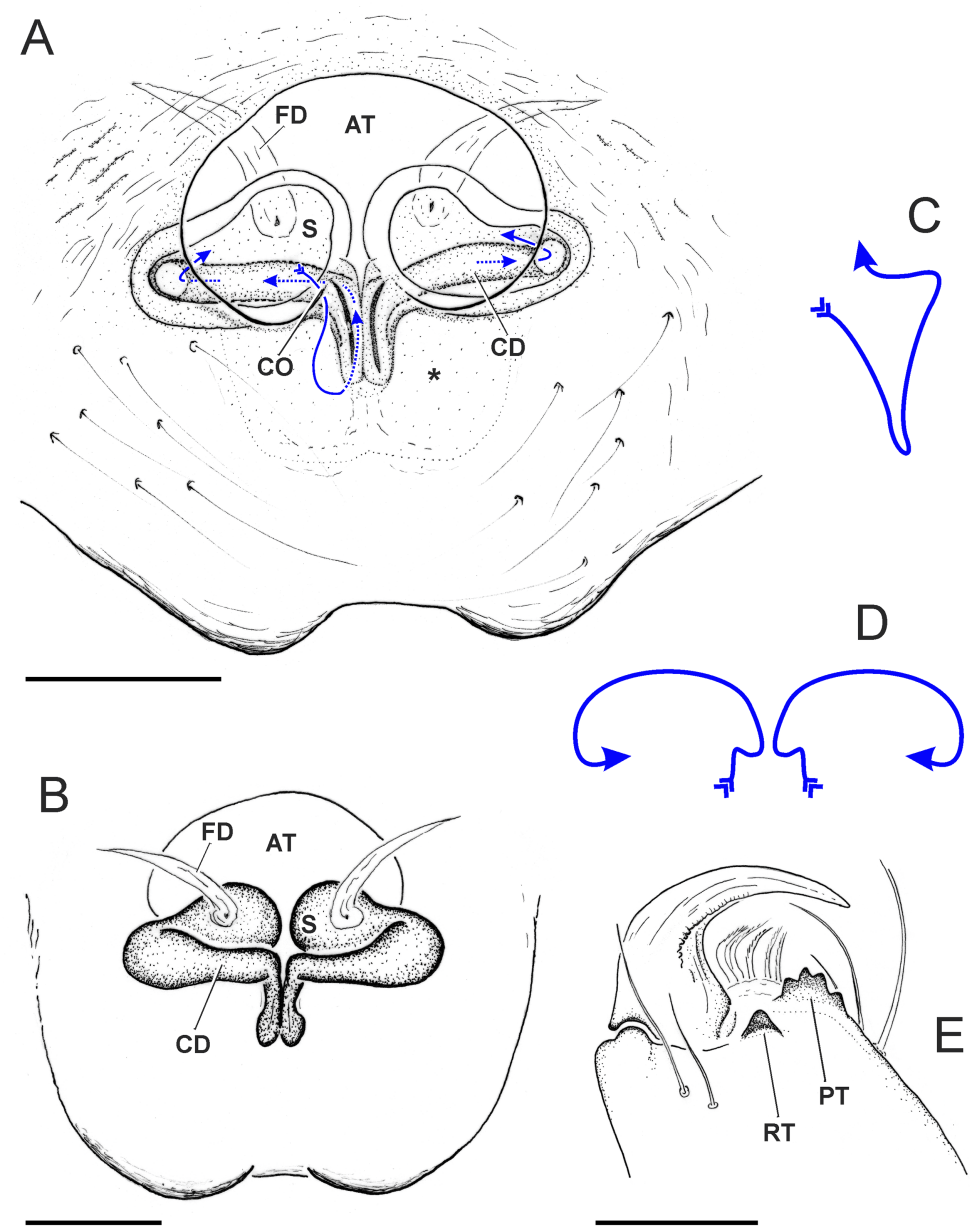
*Scoturius dipterioides* sp. nov., holotype female. A. cleared epigyne in ventral view (blue line course of CD). B. ditto, dorsal view (vulva). C. course of CD in left lateral view. D. ditto, anterior view. E. right chelicera in posterior view. AT = atrium. CD = copulatory duct. CO = copulatory opening. FD = fertilization duct. S = spermatheca. Scale bars: 0.1 mm.

**Diagnosis**. *Scoturius* can be distinguished from other Huriini, except *Urupuyu* Ruiz & Maddison, 2015, based on their possessing a large anterior elliptical opening (forming an atrium) with the initial portion of the copulation ducts dilated as a chamber (Fig 2A; compare with [17]: Figs 25 and 26), and can be demarcated from *Urupuyu* in their having more joining at the copulation openings and shorter copulation ducts (Fig 2A and 2B; compare with [17]: Figs 25 and 26).

***Scoturius dipterioides* sp. nov**. urn:lsid:zoobank.org:act:0F2706BF-1EA3-425C-A6C9-78E380C6E9E

**Fig 3:**
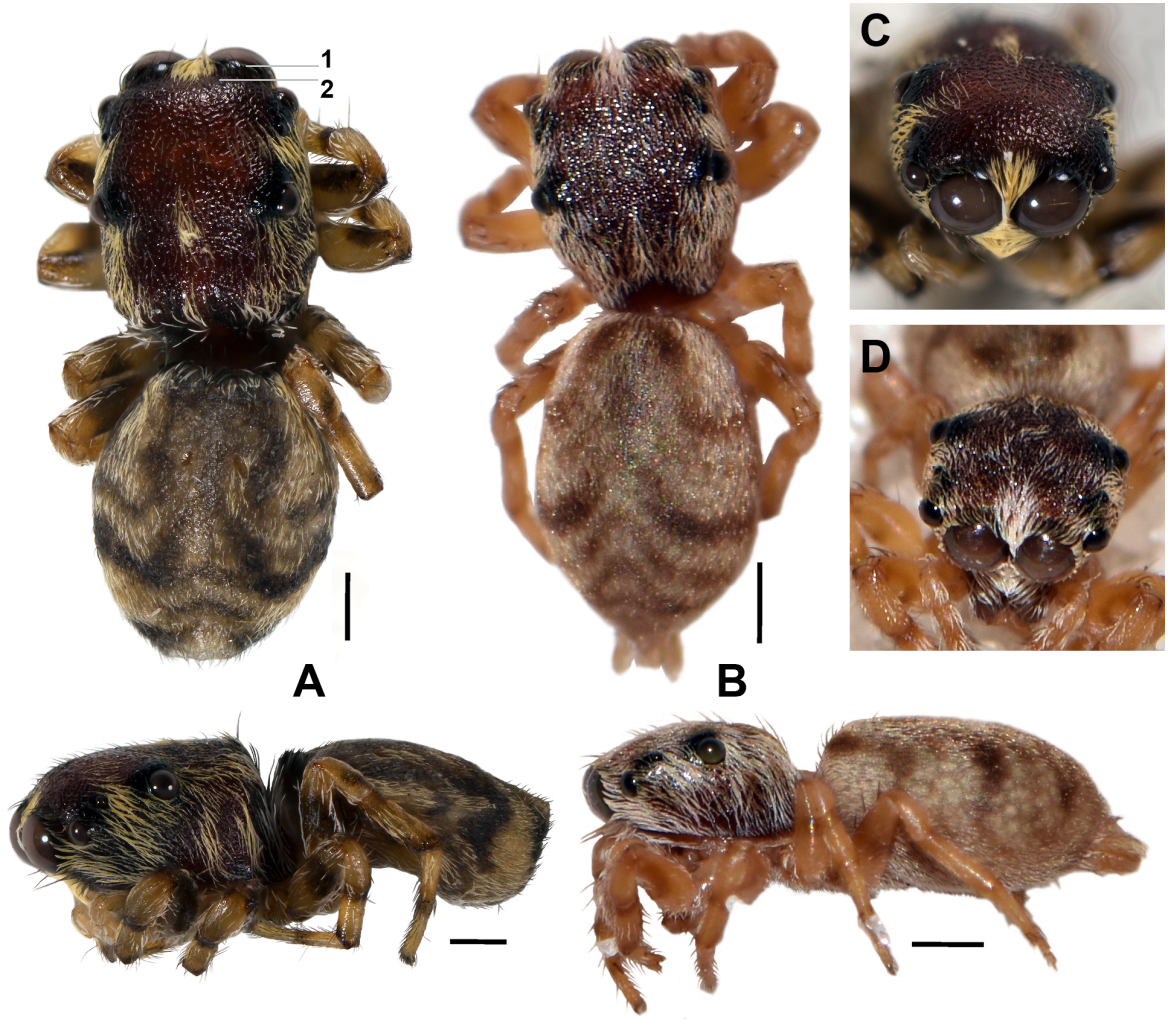
Dorsal and lateral habitus of females, dry condition, life color. A. *Scoturius dipterioides* **sp. nov**., female holotype; characteristics that increase fly-like appearance and are not found in other species of the Huriini: 1 = the border of the anterior median eyes having same color as the latter and sparse, indistinct setae, giving the impression that the anterior median eye and its border form a single, larger unit (imitating large fly eyes). 2 = the cephalic area is slightly edged shortly behind the anterior border (imitating the anterior border of fly pronotum). B. *Atelurius segmentatus* Simon, 1901 (please note that the border of the anterior median eyes is covered by light setae). Dorso-frontal view: C. *S*. *dipterioides* **sp. nov**., holotype. D. *A*. *segmentatus*. Scale bars: 1 mm.

**Fig 4:**
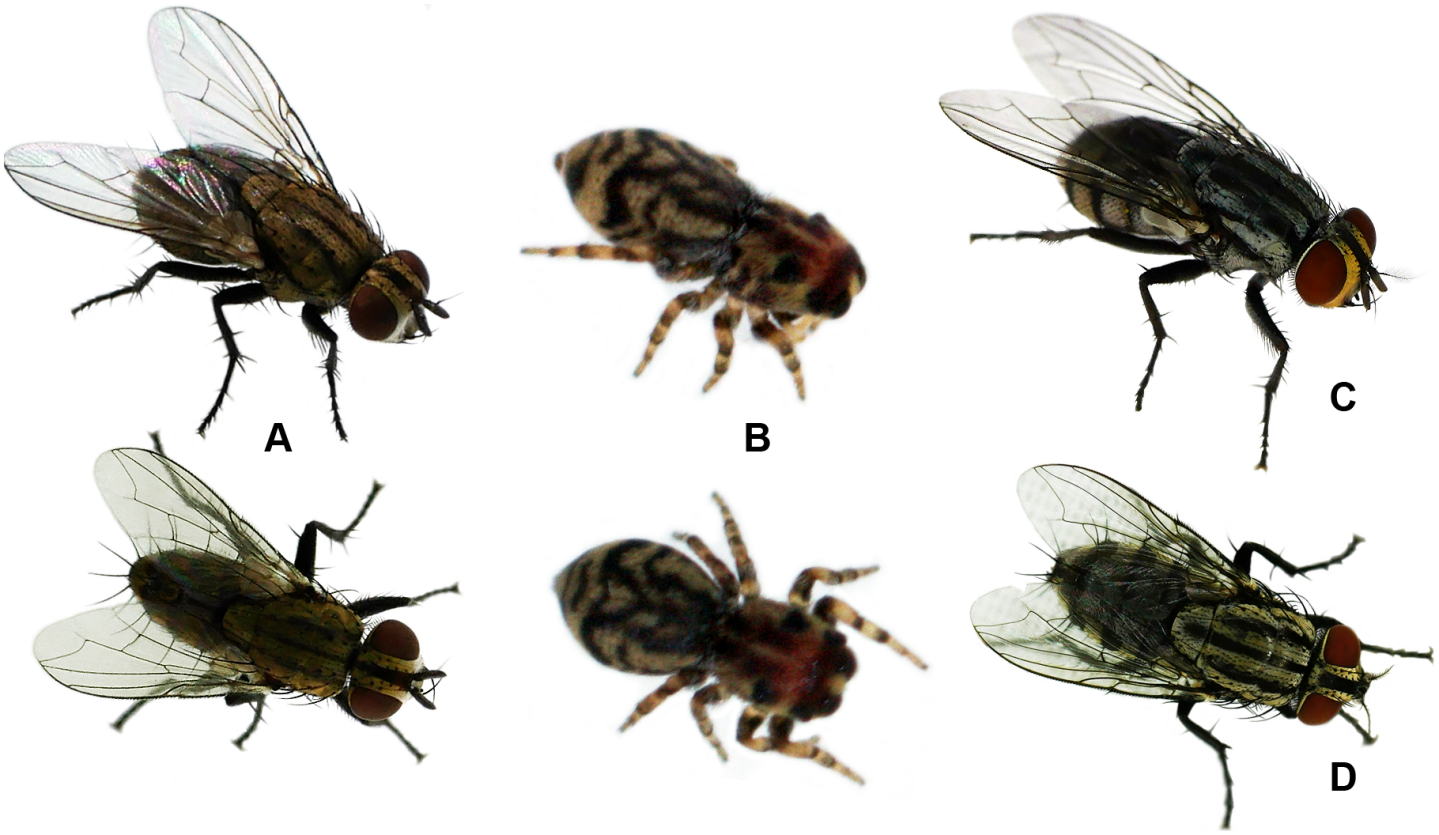
Possible fly models (A. same species. C. and D. different species) of *S*. *dipterioides* sp. nov. (B. life habitus of holotype); fly species not determined, body length ~4–6 mm.

**Fig 5:**
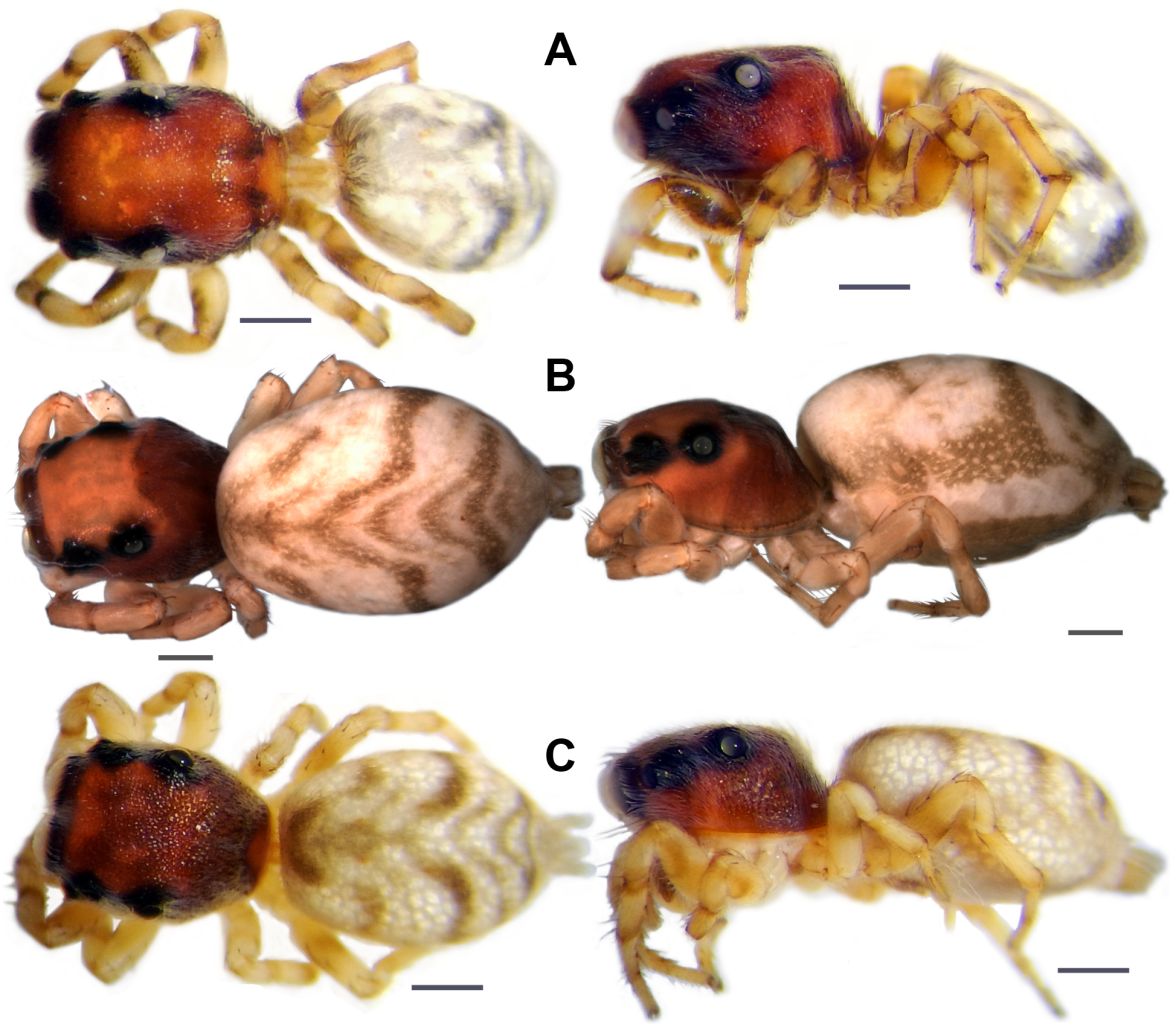
Dorsal and lateral habitus after more than 18 months preservation in ethanol (80%). A. *Scoturius dipterioides* **sp. nov**. B. *Atelurius segmentatus* Simon, 1901. C. *Scoturius tigris* Simon, 1901. Scale bars: 1 mm.

Figs 2, 3A, 3C, 4B and 5A

**Type material**. Holotype female from BOLIVIA, *Santa Cruz department*: La Guardia (Chiquitano forest; 17°52’59”S / 63°19’04”W; 480 m a.s.l.), XI-XII.2015, beating tray, R. Perger col. (IBSI-Ara 0713; tiss.s.GDR 0920).

**Etymology**. The species name refers to the species’ resemblance to flies.

**Diagnosis**. *Scoturius dipterioides* **sp. nov**. can be distinguished from *S*. *tigris* by the kidney shaped spermathecae (spherical in *S*. *tigris*), situated at the atrium level (more backwards in *S*. *tigris*) (Fig 2A and 2B; compare with [16]: Figs 7 and 8).

**Description**. Female (holotype). Total length: 3.90. Carapace 1.84 long, 1.22 wide, 0.80 high. Clypeus low, 0.12. Ocular quadrangle 0.72 long. Anterior eye row 1.16 wide and posterior 1.22 wide. Length of femur I: 0.70, II: 0.67, III: 0.70, IV: 0.95; patella + tibia I: 0.82, II: 0.75, III: 0.72, IV: 0.92; metatarsus + tarsus I: 0.70, II: 0.62, III: 0.77, IV: 0.85. Leg spination: femur I–III d 0-0-2(1p), IV d 1-1-2(1r); patella I–IV 0; tibia I v 0-0-1p, II-III 0, IV r 1; metatarsus I v 2–2, II v 0-1r-1p, III v 0-0-1p, d 0-0-1r, IV v 0-0-1, p 0-0-2(1v); tarsus I–IV 0. Leg formula 4132. Cephalothorax moderately high, lateral borders slightly convex, narrowing anteriorly and posteriorly when seen in dorsal view, cephalic part as wide as thoracic part; narrowing towards AME when seen in lateral view; coarsely wrinkled. Quadrangle of eyes almost as twice as wide as long, slightly wider behind than in front, occupying about the middle of cephalothorax. Cephalic area slightly edged shortly behind anterior border. Chelicerae small, vertical, promargin with four teeth, retromargin with one tooth. Epigyne (Fig 2A and 2B): epigynal plate conspicuous, posterior border indented, with a large anterior elliptical opening forming an atrium which leads to two copulation ducts begin as wide chambers (hard to see). Copulatory ducts short, extend backwards, sideways and entering the spermathecae dorsally. Spermatheca kidney-shaped; fertilization duct anterior to spermatheca.

Colour and setation (Fig 3A and 3C): Chelicerae dark-brown, with single appressed white setae. Border of AME having same color of AME and sparse, indistinct setae that do not obscure integument, giving impression that AME and border form single, larger unit. AME and chelicerae separated bilaterally by band of appressed cream setae. Cephalothorax reddish, anterior half black laterally, black area terminating at a hypothetical line between ALE and PLE; cephalothorax laterally to dorsolaterally with an area of cream setae, area with two vertical bands with darker, sparser setae parallel to posterior border of cephalothorax (only visible when seen in lateral view); cephalothorax dorso-median with longitudinal row of cream setae, terminating at height of PME; transversal area along anterior dorsal border of cephalic part without setae. Abdomen dark-grey/brownish, proximal two-third with grey/dark-yellow wing-shaped pattern furnished with whitish-grey setae, distal third with three transversal grey/dark-yellow bands that are furnished with whitish-grey setae, the anterior two bands connected laterally. Legs beige to dark yellow, distal borders of segments dark brown, femora median with brown-greyish patches.

**Remarks**. The mature male of *Scoturius dipterioides* **sp. nov**. remains unknown. As extensive sampling in the study area only revealed one female and one immature male, and seeing that it is uncertain when additional material will be collected, we decided to propose the new taxon based on the available specimens. The new species was tentatively placed into *Scoturius* because the epigynum has a single large elliptical opening. Further studies including the mature male may indicate that this species belongs to another (maybe new) genus. The resemblance of *S*. *dipterioides* **sp. nov**. to flies is attributed to at least nine characteristics (Table 1; Figs 3A, 3C and 4). The reddish cephalic area in combination with a grey-blackish pattern on the body suggests that *S*. *dipterioides* **sp. nov**. is a mimic of smaller calyptrate muscoids (see Fig 4). However, there are also acalyptrate flies that exhibit these colour patterns, found in Bolivia and may signify models for *S*. *dipterioides* **sp. nov**., for example species of the genera *Eurystratiomyia* and *Physegeniopsis* (Lauxaniidae) (D. Whitmore, pers. comm.; see [22]), or *Cyphomyia* (Stratiomyidae) (H. Hespenheide, N. E. Woodley, pers. comm.).

**Table 1:** Morphological characters increasing the resemblance of *Scoturius dipterioides* sp. nov. to flies with grey/blackish patterns; specific characters with bold numbers.

Fly characters	*Scoturius dipterioides* sp. nov.	No.
Large eyes	border of AME having same color as AME and sparse, indistinct setae, giving impression that AME and border form single, larger unit	**1**
Red eyes	cephalothorax reddish anteriorly	2
Whitish areas on frontorbital plate, parafacialia and proboscis	AME and chelicerae separated by longitudinal band of cream setae, respectively	3
Pronotum	cephalic area slightly edged shortly behind anterior border	**4**
	Transversal, dark band behind anterior border of cephalic part without hairs, resembling to anterior pronotal border	5
	ALE imitating antero-lateral edges of pronotum	6
Longitudinal stripes on pronotum	longitudinal bands of light setae on dorsal cephalothorax	7
Wings	light wing shaped pattern on abdomen, furnished with light setae	8
Segmented abdomen	transversal dark and light stripes, both furnished with setae of similar color	9

The live colour has not been described for most species of the Huriini and in the current study, fresh specimens were available only for *Scoturius dipterioides* **sp. nov**. and *Atelurius segmentatus* Simon, 1901 (the colour fades in preserved material). However, a reddish cephalic part (characteristic 2) and patterns that may be interpreted as imitations of wing patterns (characteristic 8) and abdominal segmentation (characteristic 9) are also present in museum specimens of *A*. *segmentatus* and *Scoturius tigris* (see Fig 5). Subsequent investigations may reveal that the colour of additional species resembles that of flies.

Two of the nine characteristics that increase resemblance to flies are not present in other species of the Huriini (cephalic area edged anteriorly and a difficult to view separation between the anterior median eyes and their borders, see Table 1; Figs 3 and 5) and render *Scoturius dipterioides* **sp. nov**. the most fly-resembling form of this tribe.

## Discussion

*Scoturius dipterioides* **sp. nov**. is the first species of the Huriini that is reported for Bolivia. This discovery is not surprising as the tribe is present from Colombia to Argentina and Chile [3] and the knowledge of spiders in Bolivia is strongly influenced by sampling bias [23,24], which is in agreement with the poor data of other arthropods [25–29]. However, judging from the low abundance of *S*. *dipterioides* **sp. nov**., despite the great sampling effort (2 individuals/~100 hrs sampling), and the lack of representatives of this tribe in numerous spider samples that were obtained from other ecoregions in Bolivia (Perger, data not shown), the little in the way of knowledge may also result from a cryptic way of life or the difficult accessibility of the putative habitat of the Huriini (e.g., forest canopy).

### Fly mimicry

Given that previous observations of fly mimicry in spiders [13] have not been confirmed by more recent studies [14,15], *S*. *dipterioides* **sp. nov**. is the only spider taxon that is currently suggested to mimic flies. The accuracy of fly resemblance in the newly described species, ascribed to modifications in body shape (cephalic area edged anteriorly and an indistinct separation between the anterior median eyes and their borders) that are not found in other species of this tribe (Table 1), may imply that *S*. *dipterioides* **sp. nov**. represents an advanced stage in the evolution of fly mimicry. On the other hand, the colour patterns of certain species (e.g., *Atelurius segmentatus* and *Scoturius tigris*, see Fig 5) may signify precursors of fly mimicry.

Despite the conspicuousness of a fly-like appearance [1,29,30], its evolutionary advantage remains unclear and avoidance by potential predators has not been demonstrated by empirical data [2]. The existence of flies that imitate jumping spiders [31] and jumping spiders that resemble to flies (as in this study) indicate a complex pattern of mimicry selection, probably involving various types of predator regimes. Like what was suggested for fly-resembling beetles [1], a possible benefit from possible fly mimicry in the Huriini could be that visually-oriented predators, like birds and jumping spiders, may sometimes decline the opportunity to pursue fast-flying flies. Furthermore, the assumed fly mimicry may be advantageous in the presence of predators that have a narrow diet breadth (stenophagy) and are specialized on prey groups other than dipterans. Pekár et al. [32] found that the species diversity of stenophagous genera and families among 587 spider species was similar to euryphagous genera and families. Apart from dipterophagous predators, stenophagous groups included myrmecophagous, araneophagous and lepidopterophagous spider taxa. Particularly in presence of araneophagic spiders, such as species of the jumping spider genus *Colonus* (tribe Gophoini) [33], fly mimicry may be beneficial in spiders. In the investigated forest fragment, *Colonus germaini* (Simon, 1900) was among the most abundant salticids in midstorey vegetation (Perger, unpubl. data) and could be a possible agent for the selection of fly mimicry.

Another mechanism selecting for fly mimicry in spiders may be an easier approach and capture of prey because the latter likely considers flies less dangerous than spiders. Mimicking other organisms to access resources or prey (occasionally the model itself) is recognized as “aggressive mimicry” [34] and has been observed in ant-mimicking salticids that prey upon their models [34] or araneophagic salticids that invade webs and manipulate silk lines to imitate courting males or ensnared insects [35]. In *S*. *dipterioides* **sp. nov**., the advantage of increased capture rates may outweigh the possibility that the fly-resembling spider attracts other, dipterophagous jumping spiders.

Further sampling in other habitats will hopefully uncover a greater number of individuals of *S*. *dipterioides* **sp. nov**. to test the benefit of fly resemblance in jumping spiders in laboratory experiments.
